# Early Identification of COVID-19 Infection Using Remote Cardiorespiratory Monitoring: Three Case Reports

**DOI:** 10.2196/27823

**Published:** 2021-06-16

**Authors:** Michael Polsky, Neema Moraveji

**Affiliations:** 1 Pulmonary Associates of Richmond North Chesterfield, VA United States; 2 Spire Health San Francisco, CA United States

**Keywords:** COVID-19, remote patient monitoring, wearable sensors, monitoring, case study, preidentification, lung, data collection, respiration, prediction

## Abstract

**Background:**

The adoption of remote patient monitoring (RPM) in routine medical care requires increased understanding of the physiologic changes accompanying disease development and the proactive interventions that will improve outcomes.

**Objective:**

The aim of this study is to present three case reports that highlight the capability of RPM to enable early identification of viral infection with COVID-19 in patients with chronic respiratory disease.

**Methods:**

Patients at a large pulmonary practice who were enrolled in a respiratory RPM program and who had contracted COVID-19 were identified. The RPM system (Spire Health) contains three components: (1) Health Tags (Spire Health), undergarment waistband-adhered physiologic monitors that include a respiratory rate sensor; (2) an app on a smartphone; and (3) a web dashboard for use by respiratory therapists. The physiologic data of 9 patients with COVID out of 1000 patients who were enrolled for monitoring were retrospectively reviewed, and 3 instances were identified where the RPM system had notified clinicians of physiologic deviation due to the viral infection.

**Results:**

Physiologic deviations from respective patient baselines occurred during infection onset and, although the infection manifested differently in each case, were identified by the RPM system. In the first case, the patient was symptomatic; in the second case, the patient was presymptomatic; and in the third case, the patient varied from asymptomatic to mildly symptomatic.

**Conclusions:**

RPM systems intended for long-term use and that use patient-specific baselines can highlight physiologic changes early in the course of acute disease, such as COVID-19 infection. These cases demonstrate opportunities for earlier diagnosis, treatment, and isolation. This study supports the need for further research into how RPM can be effectively integrated into clinical practice.

## Introduction

Early identification of acute clinical deterioration can lead to proactive intervention and a reduction in morbidity in patients with or without chronic disease [1-3]. There are a number of reasons why patients may receive delayed medical care [4]. For example, patients may not recognize a change in their symptoms or may avoid contacting their provider so as not to burden themselves or the practice. The COVID-19 pandemic also appears to have exacerbated patient reluctance to seek care [5].

Early diagnosis of COVID-19, the disease resulting from SARS-CoV-2 infection, can lead to proactive management, such as increased monitoring [6] and daily prone positioning [7], as well as early patient isolation. For the novel monoclonal antibody therapies to be effective, it appears that they must be used before the patient develops severe illness [8].

Studies using data from consumer activity trackers have been reported to identify signs of COVID-19 infection before symptoms develop [9-11]. Despite the promise of medical-grade remote patient monitoring (RPM), physicians and health care organizations can be slow to adopt it. Valid reasons for this lag in acceptance include limited clinical data [12] and discomfort with unfamiliar technology [13]. Deployment of new technology can also be delayed due to a lack of technological infrastructure and integration into clinical workflow [14]. This report motivates study into how to effectively integrate RPM into clinical practice by describing three cases of patients with COVID-19 where physiologic changes were identified through RPM prior to their presentation to a medical practice.

## Methods

The RPM system (Spire Health [15]) was studied, designed, and validated for long-term use with patients with chronic respiratory disease [16,17], and it contains three components: (1) Health Tags (Spire Health), undergarment waistband-adhered physiologic monitors that require minimal patient management and include a respiratory rate sensor; (2) an app on an in-home, stationary internet-connected device (a Nokia smartphone) configured to automatically collect and upload sensor data to the cloud; and (3) a web dashboard at the Spire Health website [15] that is monitored 7 days per week by respiratory therapists (RTs) who proactively engage patients by telephone in the event of significant changes in adherence, respiratory rate, pulse rate, or activity level. The dashboard notifies the RTs of significant patient-specific deviations in respiratory metrics, pulse rate, and activity. The notifications compare each patient’s current metrics with their respective historical baseline. The system was designed to identify deviations associated with exacerbation of chronic respiratory disease.

A US-based pulmonology practice offered RPM to chronic patients with respiratory disease and had not yet defined a clinical workflow for using RPM with patients with COVID-19. At the time of review, from approximately 1000 patients enrolled in the monitoring, 9 were confirmed to have contracted COVID-19. The RPM data of these 9 patients were retrospectively reviewed and evaluated based on whether the RPM had notified clinicians of physiologic deviation around infection onset. No evaluation of the predictive performance of the RPM was performed. Three case reports demonstrated differentiated clinical cases, and the patients gave consent to use of their data.

## Results

### Case 1: Symptomatic

**Figure 1:**
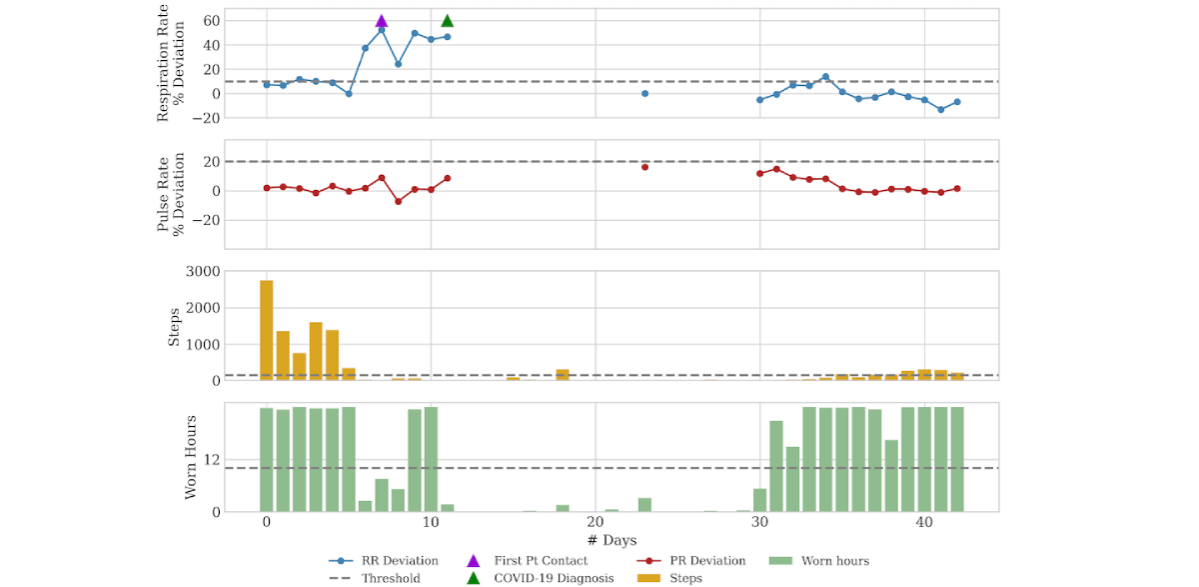
Daily physiology, activity, and adherence metrics for Case 1. The patient was hospitalized for COVID-19–related symptoms 5 days after she was contacted by the respiratory therapist call center. Physiology shows return to baseline after discharge 17 days later. Each point for RR and PR represents the percent difference between that day’s median value and that patient’s lifetime median baseline value. The thresholds are clinician-configurable points at which notifications are triggered. Default threshold values (for all three cases): worn hours <11 hours, RR % deviation: >10, PR % deviation: >20, and steps: <150. PR: pulse rate; Pt: patient; RR: respiratory rate.

Patient 1 is a 70-year-old woman with moderate chronic obstructive pulmonary disease (COPD) who had been receiving routine follow-up care. For the first 3 months of monitoring, she demonstrated stable parameters in her respiratory rate and heart rate as well as typical variations in her activity levels. Approximately 3 months prior to her next scheduled office visit, the patient was noted to have an acute increase in her respiratory rate accompanied by reduced step counts (see Figure 1). These physiologic changes triggered a notification in the monitoring system, leading to telephone contact of the patient. The patient reported feeling generally poor and attributed her symptoms to back pain. Further query by the RT call center staff did elicit increased shortness of breath and cough. Although the patient declined a pulmonary clinic visit, she was encouraged to monitor her symptoms and contact her physician or emergency department (ED) if her condition did not improve. The patient’s respiratory rate remained elevated 5 days after the initial notification, and the she presented to the ED. She was diagnosed with COVID-19 and spent 17 days in the hospital before recovering to near baseline and returning home. Her respiratory physiologic parameters returned to baseline 20 days later. Her step counts were noted to return to baseline approximately 1 month after hospital discharge.

### Case 2: Presymptomatic

**Figure 2:**
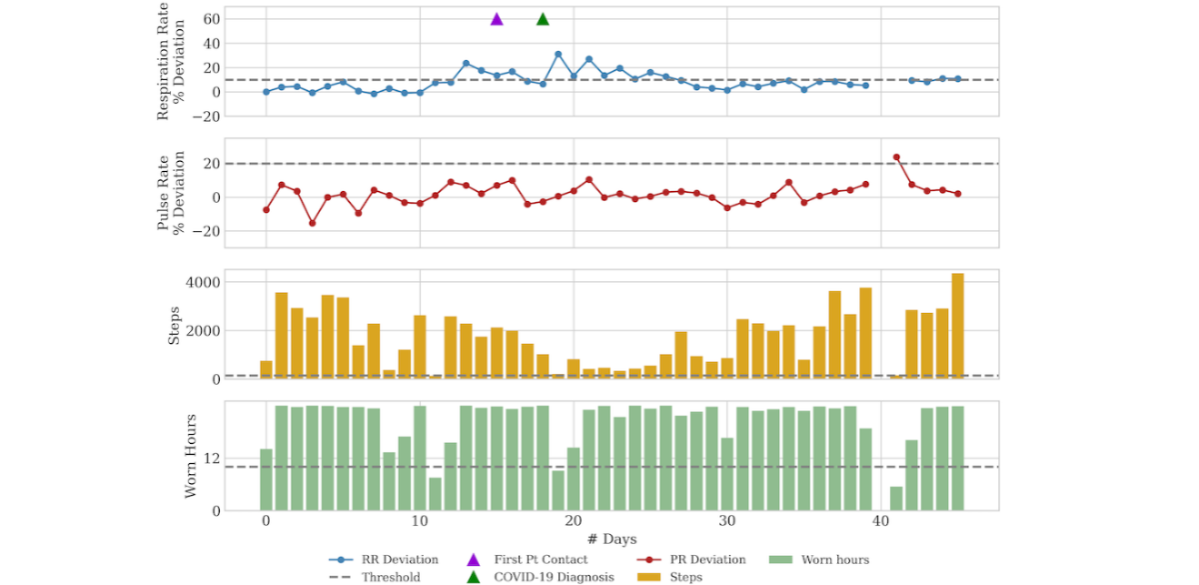
Daily physiology, activity, and adherence metrics for Case 2. COVID-19 diagnosis was confirmed 3 days after the patient was contacted and 5 days after initial remote patient monitoring notification. PR: pulse rate; Pt: patient; RR: respiratory rate.

Patient 2 is a 72-year-old man with a history of idiopathic pulmonary fibrosis. After 6 months of stable physiologic parameters, the RPM triggered a notification of a respiratory rate increase 24% above baseline (see Figure 2). The patient was reached by telephone within 48 hours of the notification, and he reported no concerning symptoms. He declined further clinical evaluation at that time. Five days after the initial notification, the patient developed body aches and a general feeling of being unwell. That day, a COVID-19 test performed by the patient’s primary care physician gave a positive result. The patient’s physiology returned to baseline 15 days after infection onset.

### Case 3: Asymptomatic to Mildly Symptomatic

**Figure 3:**
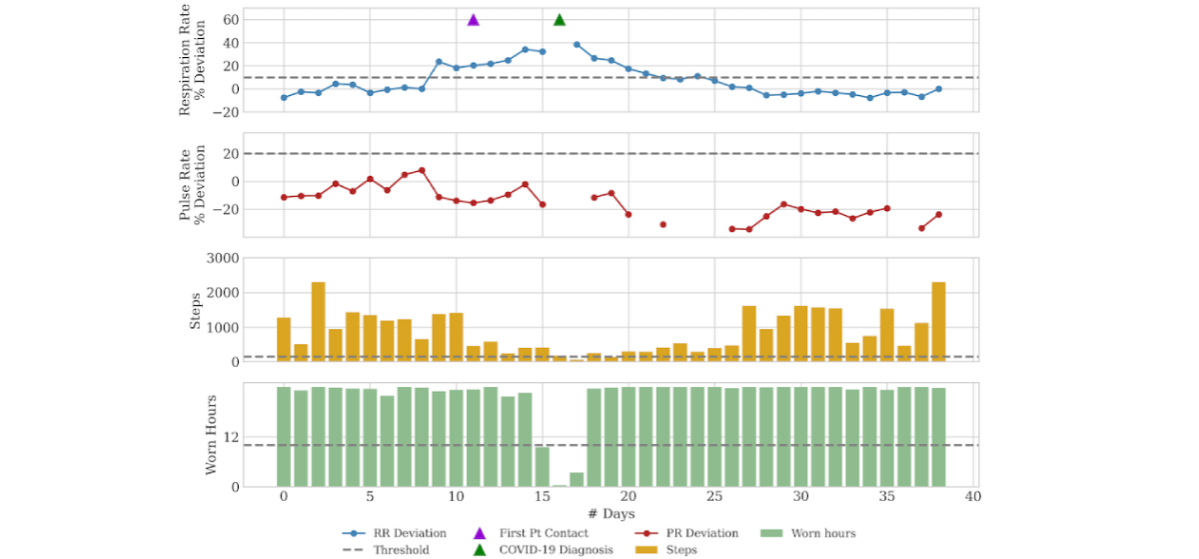
Daily physiology, activity, and adherence metrics for Case 3. Initial patient outreach based on RPM notification occurred 4 days before COVID-19 diagnosis. PR: pulse rate; Pt: patient; RR: respiratory rate.

Case 3 is an 80-year-old man with moderate COPD who had started in the practice’s remote monitoring program approximately 1 month prior to the first notification. The RPM reported a 24% increase in the patient’s respiratory rate; the patient was reached by telephone the next day and reported mild allergy-type symptoms. He opted for over-the-counter symptomatic treatment and also declined a clinic visit, as he had been seen in a clinic only 8 days prior. His respiratory symptoms remained mild 7 days after the initial notification; however, he was hospitalized for an unrelated reason. As part of hospital routine during the viral pandemic, he was tested and found to be positive for COVID-19 (see Figure 3). The patient’s physiology returned to baseline 14 days after infection onset.

## Discussion

Case 1 demonstrates the capability to identify and engage patients earlier in the course of acute disease than would otherwise be possible without remote monitoring. Although the patient declined to see her provider at the time of the telephone call, she was notified of the physiologic changes and encouraged to seek early evaluation. Delayed care in COVID-19 leads to worse outcomes, and this is particularly relevant for patients with comorbid conditions [18,19].

Unlike case 1, the second patient denied having symptoms at the time of initial notification. For this reason, he declined further evaluation. It is known that physiologic changes can occur prior to patient-reported symptoms and recognition of COVID-19. Understanding the potential significance of physiologic changes, particularly during a viral pandemic, can lead to earlier diagnosis. Home-based and rapid COVID-19 testing should be deployed liberally during a pandemic to enable early identification and patient isolation [20]. As with case 1, this patient is over 65 years old and has comorbidities. These patients require increased monitoring when diagnosed with COVID-19, and current evidence supports the consideration of monoclonal antibody therapy prior to needing hospital-level care [8].

Case 3 demonstrates a patient with mild symptoms who was incidentally found to test positive for COVID-19. It is possible that he would have recovered without knowing he had been infected with COVID-19. Similar to the prior cases, significant time passed between physiologic identification and confirmed COVID-19 diagnosis. The exact burden of asymptomatic and presymptomatic spread of COVID-19 is uncertain but is felt to be significant [21]. Even if this patient makes a full recovery, the exposure to others prior to diagnosis has implications on pandemic control. During a viral pandemic, a high index of suspicion for infection must exist in patients who demonstrate signs of infection, even in the absence of significant symptoms or concerns.

In all three instances, COVID-19 was diagnosed 5 to 7 days after the initial notification. Optimally, the physiologic notifications and high suspicion of COVID-19 related to the RPM findings would prompt earlier diagnostic testing. However, patients’ rationalization of symptoms and hesitancy to be evaluated factor into the delay [4]. Likewise, physicians may be less apt to intervene in cases where patient symptoms are minimal. Although these cases were selected by the authors, we suspect this delay in diagnosis is typical in most medical practices for the stated reasons.

One of the primary potential benefits of RPM is that deterioration can be treated earlier and more effectively. To increase RPM acceptance, data demonstrating improved patient outcomes is necessary. The success of RPM in providing these benefits is dependent on three requirements: the physiologic data is accurate, notifications are set at clinically significant levels, and medical interventions are effective and instituted in a timely manner. Continuous physiologic monitoring has shown itself to be accurate in patients with and without chronic disease [16,17]. There is emerging evidence regarding what physiologic changes from patient specific baselines on RPM are significant for various diseases, including COVID-19 [11].

The optimization and timing of medical interventions is less clear. With the expanded role of telemedicine, we propose a standardized short term clinical assessment after RPM notification, performed from the patient’s home, with a low threshold to test for COVID-19. Further research is necessary to determine if this protocol alone would be sufficient to result in the desired clinical benefit.

### Conclusion

This report suggests a blueprint in the approach to using RPM of patients with chronic respiratory disease during a viral pandemic. In these three cases, early physiologic changes secondary to COVID-19 infection detected using RPM were readily identified and patients were proactively engaged. Further research refining RPM use in clinical practice may lead to earlier diagnosis, isolation, and treatment.

## Abbreviations

COPD: chronic obstructive pulmonary disease
ED: emergency department
RPM: remote patient monitoring
RT: respiratory therapist
